# Ophthalmology as a career choice among medical students: a survey of students at a Canadian medical school

**DOI:** 10.1186/s12909-022-03295-w

**Published:** 2022-04-01

**Authors:** Bo Li, Evan Michaelov, Ryan Waterman, Sapna Sharan

**Affiliations:** 1 Department of Ophthalmology, Schulich School of Medicine and Dentistry, Ivey Eye Institute, Western University, London, ON Canada; 2 Schulich School of Medicine and Dentistry, Western University, London, ON N6A 5C1 Canada

**Keywords:** Survey, Ophthalmology, Medical students, Medical education

## Abstract

**Background:**

There is a lack of investigations into the factors that lead medical students to pursue increasingly competitive post-graduate training programs. We sought to determine the factors that influence medical students’ opinions on ophthalmology as a career and on ophthalmological medical education.

**Methods:**

An anonymous 36-question survey was distributed to all medical students across the four program years at the Schulich School of Medicine and Dentistry as a non-probabilistic convenience sample. Survey results were analyzed using Mann-Whitney U tests to determine significant differences between study sub-populations. Multivariate regression analysis was performed to identify correlates for positive views towards ophthalmology.

**Results:**

81% of questions had a mean positive response amongst the students. Students held negative views regarding the amount of exposure to ophthalmology in medical school. The greatest differences in opinion regarding ophthalmology were seen between those with more exposure and interest in ophthalmology compared to their counterparts with less. Regression analysis identified interest in ophthalmology as a significant correlate to a positive opinion in the field.

**Conclusions:**

Our survey demonstrates that while most students had positive views about ophthalmology, some aspects were viewed negatively. Students felt there was a lack of exposure, both educationally and clinically to ophthalmology, which may contribute to some misconceptions of the field. Early exposure appeared to be critical to forming positive opinions of ophthalmology and could be emphasized in medical education.

## Background

Choosing a specialty is a critical decision faced by every medical student during their medical education. In choosing a specialty, medical students are effectively choosing the future of their day-to-day-life. It is a decision that had implications that could play out over decades. Following the choice to attend medical school, it is one of the most impactful decisions in the life of a medical student. This decision is a complex one that considers several factors. In determining the focus of their postgraduate education, medical students are likely to consider a specialty’s work schedule and lifestyle, length of training, career opportunities, income, and prestige. These considerations formulate in the student’s academic interests with influence from teachers and mentors [1]. Further complicating the process is the increasing competition and declining match rates among certain specialties in the Canadian Resident Matching Service (CaRMS) [2].

To counter increasing competition, medical students are encouraged to develop a strong curriculum vitae (CV) through relevant clinical and research experiences. With limited time aside from the academic demands of medical school, students must seek out experiences in a targeted and efficient manner. Simply, there is not enough time to build a strong CV for all specialties. As a result, students must choose areas of interest early in their academic careers, or risk losing a competitive edge to other applicants.

One extrinsic factor that may play a role in students’ choice of specialty to pursue is the exposure to that subject matter in their medical school curricula. Given the finite time available, not all subjects receive the same degree of coverage during undergraduate medical courses. Smaller specialties, such as ophthalmology do not receive as much exposure as other, larger specialties [3, 4]. With less exposure to ophthalmology, it may be harder for students to discover their interest in the specialty and there may be more opportunities for misconceptions to thrive. In either case, there exists the possibility that highly qualified candidates will not be attracted to the profession.

Of interest in the following study are undergraduate medical students’ perceptions of ophthalmology as a specialty, as well as the factors underlying the development of those perceptions. While many studies have examined the factors that influence career choices in medicine, no such research has focused on ophthalmology specifically [5–7]. To determine the state of research around students’ opinions of medical specialties, a literature review was undertaken through PubMed and Google Scholar using terms including ophthalmology, perception, medical student, and undergraduate. In reviewing the existing literature, multiple publications were focused on assessing ophthalmological teaching at undergraduate and postgraduate levels [8–10]. Most of these papers focused on opinions at the post-graduate level and there was little attention to ophthalmology as a career choice. One paper by Linz et al. did address ophthalmology as a career but did so with a small target population and with a specific focus on groups underrepresented in medicine [11]. From our literature review, we determined there exists a gap in studies that assess medical students’ perceptions of ophthalmology as a destination for residency. If potential negative perceptions and misconceptions among students were better understood, it may be possible to determine directions for correction. Furthermore, while our results are centred on ophthalmology, our process may have applicability to other specialties. Our findings are specific to ophthalmology, but a similar process could be used to investigate perceptions of other specialties. Developing a better understanding of students’ perceptions in the service of seeking out the best candidates for each postgraduate program is in the best interest of all stakeholders.

To this end, we developed a survey to investigate medical students’ perception of ophthalmology as a specialty to better understand what leads students to choose or reject the specialty as a future career choice. If we can better understand what attracts students to ophthalmology, we may be able to better deliver education and information to recruit the candidates best suited for later practice.

## Methods

### Survey preparation

The survey was prepared and developed by Drs. Michaelov and Sharan on Qualtrics Software (Qualtrics, Provo, UT) and was approved by the Research Ethics Board of the University of Western Ontario before distribution (review reference 109,274–3087). Survey data was fully anonymized to maintain participant confidentiality. Consent was implied through response to the survey and participants were fully informed as to the intended use of the data. The survey was constructed using previous medical career perception surveys [12, 13], and was created in two divisions. These past surveys went through multiple iterations during a process of validation. They were trialled for comprehensiveness and appropriateness using input from medical students to determine validity. Our survey was developed based on items selected for these preceding surveys of medical student opinions. In particular, the items chosen for this survey overlap with those developed for a survey assessing choices around family medicine [5]. While the items were applied to a different specialty in that case, they were subject to a rigorous analysis to determine grouping as factors which influence career choice in medicine.

The first section was composed of 7 respondent demographic questions. These included age, gender, medical training level, the presence of a physician in the immediate family, any friends or family that were employed as ophthalmologists, level of previous exposure to ophthalmology, as well as interest in ophthalmology as a career. Previous exposure to ophthalmology was specifically defined as having a family member or friend practicing as an ophthalmologist. Beyond this specification, exposure was left to the respondent to determine if they felt they had been exposed to the profession. The second portion of the survey was composed of 36 questions related specifically to ophthalmology. This section is loosely subdivided into 6 fields covering lifestyle, culture, prestige, hospital orientation, and scope of practice of an ophthalmology career, as well as ophthalmology as a subject in medical school. The questions posed in the second portion of the survey were phrased as statements (Table 1). Participants judged the statements via 7-point Likert scale, with 1 representing “strongly disagree”, and 7 representing “strongly agree.” The intervening choices represented opinions between the extremes. Questions that were deemed as subjective were not included in the regression analysis regarding the overall opinion of the specialty.

**Table 1:** Survey Questions

Q1 What is your age? Q2 What is your gender? Q3 What level of medical training were you at during the 2016/17 academic year? Q4 Do you have an immediate family member (sibling, parent) that is a physician? Q5 Do you have a relationship (family member, friend, etc.) that is an Ophthalmologist? Q6 Have you had any previous exposure to Ophthalmology? Q7 Do you have any interest in Ophthalmology as a career?	
Q1 *The work hours required for a career in Ophthalmology are ideal. Q2 *Ophthalmology affords a very flexible career within medicine (many different scopes of practice available within the field). Q3 *Ophthalmology affords personal flexibility outside of medicine (hobbies, etc.). Q4 *An Ophthalmologist has a good lifestyle compared to other medical specialties. Q5 *Ophthalmologists are very satisfied with their careers. Q6 *The residency length required for Ophthalmology is optimal. Q7 *The lifestyle afforded to Ophthalmology residents would be considered good overall. Q8 Ophthalmology has an appropriate distribution of both male and female staff. Q9 Ophthalmologists, in general, are approachable individuals for students. Q10 Ophthalmologists, in general, are approachable individuals for patients. Q11 Ophthalmology has an appropriate distribution of both male and female residents. Q12 Ophthalmology residents, in general, are approachable individuals for students. Q13 Ophthalmology residents, in general, are approachable individuals for patients. Q14 *Ophthalmologists are able to create long-term relationships with patients in their practice. Q15 Ophthalmologists serve a diverse patient population. Q16 *Ophthalmology patients are often very satisfied with their care and results from treatment. Q17 Ophthalmology is a very competitive specialty to enter. Q18 *Ophthalmology is seen as a prestigious specialty amongst physicians. Q19 Ophthalmologists are, in general, very humble. Q20 *Ophthalmologists are, in general, very intelligent. Q21 *Ophthalmology has a good mix of urgent and chronic care. Q22 *Ophthalmology has a good ratio of inpatient to outpatient care. Q23 Ophthalmology, in general, experiences quick results with their treatment interventions. Q24 *Ophthalmology has good interaction with other specialties (there are no feelings of isolation as an Ophthalmologist). Q25 *There are adequate job opportunities for graduating Ophthalmologists. Q26 *Ophthalmologists do not have a difficult practice to maintain. Q27 *Ophthalmologists have a wide range of practice. Q28 *Ophthalmologists have a substantial impact on patient quality of life. Q29 *Ophthalmologists have an excellent balance between clinic and operating room time. Q30 *There is a substantial role for research in the Ophthalmology specialty. Q31 You have had good exposure to the field of Ophthalmology in medical school. Q32 Concepts relevant to the field of Ophthalmology have been easy to grasp. Q33 You have found the Ophthalmology material presented in medical school to be interesting and stimulating. Q34 You believe the teaching you have received in regard to Ophthalmology has been relevant to your clinical experience. Q35 Ophthalmology is not an exclusive specialty and is not restricted to the health of the eye. Q36 Ophthalmology staff are generally intense personalities.	

### Survey Population

The anonymous online survey was distributed to all medical students (Year 1 to Year 4) of the 2016/17 academic year at the Schulich School of Medicine and Dentistry at Western University (London, Ontario, Canada). Distribution was completed via email and online forums. A follow-up reminder email was sent 1 week following the first to increase the response rate. Survey responses were taken between the dates of July 27, 2017, and August 18, 2017. Only fully completed surveys were included in the analysis. To briefly comment on the group of students who responded to the survey, we can infer that they have some pre-existing opinions on ophthalmology as a specialty. Participation was optional and it was made clear that the survey’s focus was opinions on ophthalmology. As such, it follows that those who participated had some degree of pre-existing opinion toward ophthalmology. However, we are limited in what inferences we can make about the perceptions of the remaining population who did respond to our survey.

### Statistical Analysis

Responses collected from Qualtrics servers were imported into SPSS (IBM) for data analysis. Survey demographics were recorded, and mean response values with standard deviation (SD) were calculated for each survey question for both overall and subgroup responses. To determine significant differences in responses of various demographic groups, Mann-Whitney U tests were performed for gender, previous exposure to ophthalmology, interest in ophthalmology as a career, whether a family member was a physician, and whether the individual had a family member or friend that was employed as an ophthalmologist. Medical students were separated as junior (undergraduate years 1 and 2) and senior (undergraduate years 3 and 4) medical students for analysis.

Regression analysis was performed with a multivariate linear regression model, as well as univariate linear regression analysis for comparison and verification of variables determined to be significant. Only a subset of questions determined to be less subjective was used for regression analysis, to best represent an appropriate evaluation of an individual’s opinion of ophthalmology. The questions used for regression analysis are denoted with an asterisk in Table 1. Survey scores used for this analysis were presented as a percentage of the highest possible score to assist with clarity.

## Results

One hundred thirty-five completed surveys were received out of 680 medical students at Western University with access to the survey, for a response rate of 20%. The full demographics can be seen in Table 2.

**Table 2:** Student demographics and characteristics (*n* = 135)

Gender (%)	
Male	76 (56.3%)
Female	58 (43.0%)
Non-Binary/Third Gender	1 (0.7%)
Age (SD)	25.34 (2.18)
Med School Year (%)	
M1	31 (23%)
M2	33 (25%)
M3	54 (40%)
M4	16 (12%)
Family Member Physician (%)	
Yes	19 (14%)
No	116 (86%)
Family or Friend Ophthalmologist (%)	
Yes	6 (4%)
No	129 (96%)
Previous Exposure (%)	
Yes	30 (22%)
No	105 (78%)
Interest in Ophthalmology (%)	
Yes	11 (8%)
No	124 (92)

SD Standard deviation

Comparison of genders demonstrated significantly higher ratings by females for the perceived level of prestige of ophthalmologists (*P* < 0.01) and the perceived level of work intensity of ophthalmologists (*p* < 0.05).

Survey responders with physician(s) as family members had significantly greater responses for simplicity of ophthalmology concepts and interest in the material (*p* < 0.04 and *p* < 0.04 respectively). Responders with family and friends who are (and/or were) ophthalmologists perceived staff physicians as having greater career flexibility (*p* < 0.03), better relationships with their patients (*p* < 0.01), and a wider range of clinical practice (*p* < 0.01). Additionally, these respondents perceived ophthalmologists as having a greater positive impact on patient quality of life (*p* < 0.05) and practising with a preferable balance of time in clinic to time in the operating room (*p* < 0.05).

Looking at senior medical students who responded to the survey, other significant findings emerge. Upper-year students perceived ophthalmology staff physicians as humble (*p* < 0.04) and highly approachable for students and patients alike (*p* < 0.01). Senior students also perceived ophthalmology residency programs to feature favourable gender distribution (*p* < 0.04). In terms of unfavourable perceptions, senior students indicated the low ratio of inpatients to outpatients as a negative aspect of the profession (*p* < 0.02).

Students with exposure to ophthalmology responded significantly higher on the Likert scale for 8 questions. These questions included staff approachability for students (*p* < 0.01) and patients (*p* < 0.01), resident approachability for students (*p* < 0.03) and patients (*p* < 0.04), the humility of ophthalmologists (*p* < 0.05), distribution of urgent and non-urgent care (*p* < 0.04), impact on patient quality of life (*p* < 0.01), and clinic to operating room balance (*p* < 0.04).

Lastly, an individual’s interest in ophthalmology as a career carried a substantial correlation with a positive response to the survey. Of students who wished to pursue ophthalmology, career flexibility (*p* < 0.05), resident approachability for patients (*p* < 0.04), patient relationships formed as an ophthalmologist (*p* < 0.01), patient diversity of an ophthalmologist (*p* < 0.01), humility (*p* < 0.05), inpatient to outpatient ratio (*p* < 0.01), range of practice (*p* < 0.02), and clinic to operating room ratio (*p* < 0.03) all scored significantly higher on average on the Likert scale than those with less interest. Full results can be seen in Tables 3 and 4.

**Table 3:** Survey responses of those with and without exposure and interest

Factor, mean (SD)	Overall	Previous exposure	No Exposure	*P*-value	Interest	No interest	*P*-value
**Lifestyle**							
Work hours^a^	5.39 (1.22)	5.43 (1.33)	5.37 (1.20)	0.653	5.64 (1.36)	5.36 (1.21)	0.307
Career Flexibility^a^	4.22 (1.61)	4.43 (1.85)	4.16 (1.54)	0.330	5.09 (1.97)	4.15 (1.56)	**0.042**
Personal Flexibility^a^	5.58 (1.16)	5.67 (1.06)	5.55 (1.18)	0.740	5.72 (1.10)	5.56 (1.16)	0.662
Lifestyle^a^	5.79 (1.14)	5.90 (1.06)	5.76 (1.16)	0.648	6.00 (1.00)	5.77 (1.15)	0.605
Career Satisfaction^a^	5.30 (1.22)	5.20 (1.50)	5.33 (1.14)	0.939	5.54 (0.82)	5.28 (1.25)	0.544
Residency Length^a^	4.67 (1.12)	4.57 (1.48)	4.70 (1.00)	0.866	4.81 (1.17)	4.65 (1.12)	0.399
Residency Lifestyle^a^	5.07 (1.32)	5.10 (1.45)	5.06 (1.28)	0.761	5.18 (1.54)	5.06 (1.30)	0.588
**Culture**							
Staff Gender Distribution	4.04 (1.10)	4.23 (1.30)	3.99 (1.03)	0.415	3.73 (1.35)	4.07 (1.08)	0.435
Staff Approachability - Students	4.47 (1.51)	5.00 (1.62)	4.32 (1.44)	**0.009**	4.91 (1.70)	4.44 (1.49)	0.241
Staff Approachability – Patients	4.75 (1.14)	5.33 (1.12)	4.58 (1.09)	**< 0.001**	5.36 (1.29)	4.69 (1.11)	0.105
Resident Gender Distribution	4.15 (0.89)	4.30 (1.21)	4.10 (0.77)	0.283	4.18 (1.25)	4.15 (0.85)	0.477
Resident Approachability – Students	4.81 (1.37)	5.23 (1.52)	4.69 (1.30)	**0.029**	5.45 (1.04)	4.75 (1.38)	0.093
Resident Approachability - Patients	4.84 (1.01)	6.17 (1.21)	4.74 (1.04)	**0.030**	5.64 (1.12)	4.77 (1.06)	**0.016**
Patient Relationships^a^	4.13 (1.43)	4.50 (1.63)	4.03 (1.35)	0.124	5.55 (1.29)	4.01 (1.38)	**0.002**
Patient Diversity	4.67 (1.38)	4.77 (1.43)	4.65 (1.37)	0.539	5.73 (1.10)	4.58 (1.37)	**0.008**
Patient Satisfaction^a^	5.17 (1.14)	5.37 (1.25)	5.11 (1.10)	0.154	5.64 (1.12)	5.13 (1.13)	0.188
Intensity	4.15 (1.05)	3.97 (1.03)	4.20 (1.06)	0.566	4.09 (1.14)	4.15 (1.05)	0.958
**Prestige**							
Competitiveness	6.46 (1.03)	6.33 (1.15)	6.50 (0.99)	0.180	6.73 (0.47)	6.44 (1.06)	0.501
Prestige^a^	5.19 (1.54)	5.00 (1.51)	5.24 (1.55)	0.360	5.09 (1.22)	5.19 (1.57)	0.541
Humility	3.42 (1.25)	3.83 (1.05)	3.30 (1.29)	**0.047**	4.27 (1.35)	3.35 (1.22)	**0.042**
Intelligence^a^	4.90 (1.13)	5.13 (1.22)	4.84 (1.10)	0.128	5.36 (1.12)	4.86 (1.13)	0.216
Role in Research^a^	5.41 (1.22)	5.57 (1.36)	5.37 (1.18)	0.293	6.00 (1.10)	5.36 (1.22)	0.081
**Scope of Practice**							
Mix of Urgent and Non-Urgent Care^a^	4.38 (1.34)	4.80 (1.47)	4.26 (1.29)	**0.033**	5.00 (1.61)	4.32 (1.31)	0.125
Inpatient-Outpatient Ratio^a^	3.71 (1.37)	3.83 (1.68)	3.67 (1.27)	0.686	5.09 (1.58)	3.59 (1.28)	**< 0.001**
Rapid Patient Results	5.19 (1.11)	5.30 (1.42)	5.15 (1.02)	0.160	5.36 (1.21)	5.17 (1.11)	0.575
Range of Practice^a^	3.90 (1.30)	3.93 (1.41)	3.90 (1.28)	0.836	4.91 (1.58)	3.81 (1.24)	**0.019**
Impact on Patient QoL^a^	5.81 (1.15)	6.27 (1.08)	5.69 (1.14)	**0.005**	6.36 (1.03)	5.77 (1.15)	0.057
Clinic – OR Balance^a^	4.73 (1.12)	5.10 (1.32)	4.62 (1.04)	**0.031**	5.55 (1.37)	4.65 (1.07)	**0.022**
**Hospital Orientation**							
Specialty Interaction^a^	3.52 (1.20)	3.60 (1.45)	3.50 (1.12)	0.839	4.00 (1.84)	3.48 (1.12)	0.271
Adequate Job Opportunities^a^	3.92 (1.30)	4.10 (1.25)	3.87 (1.29)	0.473	4.27 (1.85)	3.89 (1.24)	0.423
Practice Maintenance^a^	4.17 (1.35)	4.23 (1.33)	4.15 (1.36)	0.685	4.09 (1.64)	4.18 (1.33)	0.656
**Subject material**							
Exposure in Medical School	2.90 (1.40)	3.53 (1.60)	2.72 (1.30)	**0.012**	2.18 (0.98)	2.97 (1.41)	0.082
Concept Simplicity	4.06 (1.31)	4.33 (1.37)	3.98 (1.29)	0.245	4.36 (1.03)	4.03 (1.33)	0.409
Interesting Material	4.26 (1.42)	4.77 (1.41)	4.11 (1.40)	**0.027**	4.73 (1.56)	4.22 (1.41)	0.298
Relevance of Material	4.56 (1.30)	5.13 (1.11)	4.39 (1.31)	**0.008**	4.45 (0.93)	4.56 (1.33)	0.671
Applicability of Material	3.27 (1.32)	3.50 (1.28)	3.20 (1.33)	0.205	3.27 (1.49)	3.27 (1.31)	0.997

^a^Denotes questions used for regression analysis. *SD* Standard deviation, *QoL* Quality of life

**Table 4:** Adjusted survey scores of corresponding student characteristics (*n* = 134)

Total (SD)	68.0% (9.5%)
Gender (SD)	
Male	68.2% (10.5%)
Female	67.6% (8.0%)
Med School Year (SD)	
M1	65.5% (9.5%)
M2	70.1% (8.2%)
M3	68.6% (7.9%)
M4	65.9% (15%)
Family Member Physician (SD)	
Yes	69.7% (7.6%)
No	67.7% (9.7%)
Family or Friend Ophthalmologist (SD)	
Yes	73.3% (9.6%)
No	67.7% (9.7%)
Previous Exposure (SD)	
Yes	70.4% (12.3%)
No	67.3% (8.5%)
Interest in Ophthalmology (SD)	
Yes	75% (10.6)
No	67.3% (9.2%)

*SD* Standard deviation

Multivariate linear regression analysis included all 7 patient characteristics gathered in the first portion of the survey. No equation of significance was found, however “interest in ophthalmology” was determined to be a significant variable that affected overall opinion on ophthalmology (F (7,126) = 1.76, *p* < 0.05, with an R2 of 0.089). The average score of all students on the adjusted survey score, which included only the questions listed with an asterisk in Table 1 was 68.0 ± 9.5% of the total possible score. Multivariate regression analysis results can be seen in Table 5.

**Table 5:** Multivariate logistic regression analysis of factors associated with a positive perception of Ophthalmology

Factor	n	Standardized ß	*P* value
Age (SD)	25.34 (2.18)	−0.120	0.196
Gender (Male, %)	76 (56.3%)	−0.020	0.827
Medical School Year (Senior, %)	70 (52.2%)	0.121	0.214
Family Member Physician (%)	19 (14%)	0.042	0.628
Family or Friend Ophthalmologist (%)	6 (4%)	0.110	0.212
Exposure (%)	30 (22%)	0.081	0.360
Interest (%)	11 (8%)	0.218	**0.016**

*SD* Standard Deviation

As a surrogate for the students’ opinion of ophthalmology, simple linear regression analysis predicted interest in ophthalmology as having a significant relationship (F (1,132) = 6.782, *p* < 0.011) with the adjusted survey score, with an R2 of 0.049. The expected survey score increases by 7.6% when the student is interested in ophthalmology as a career. No other simple linear regression analysis revealed any significant relationships.

Most students felt positive about ophthalmology as a specialty in general, as indicated by more than 80% of respondents who selected a positive statement on the 7-point Likert scale (a score of 5, 6, or 7). Aspects of ophthalmology that were particularly well-perceived by the study population included personal flexibility and lifestyle.

## Discussion

Ophthalmology is often perceived as one of the more flexible surgical specialties amongst the medical community [11]. However, according to previous Canadian Medical Association (CMA) surveys, which is a resource often frequented by medical students when looking into potential career choices, an ophthalmologist’s workweek, excluding call, sits at approximately 50.2 h. This is comparable to surgical specialties such as general surgery (54.3 h) and orthopedics (50.9 h) [5]. Call commitments of ophthalmologists also appear to be more burdensome than currently perceived by students, with up to 25% of physician respondents reporting being on-call between 120 and 240 h every month [14].

However, these statistics may be misleading due to several factors. First of which would be the difference between subspecialties within the field of ophthalmology. For example, a retina specialist may be expected to be on call more than a general ophthalmologist, depending on the coverage schedule in the area. Furthermore, academic and community practices should be differentiated. While community physicians may be tasked with call for a greater amount of time, it is likely they would have a much less onerous patient load. Further discussion with students on the expectations of a career in ophthalmology would facilitate a better understanding of the ophthalmologist’s duties and work commitments.

Survey results were assessed to determine which characteristics influenced certain perceptions of ophthalmology as a career. The least influential characteristic appeared to be gender, which had significant differences in responses on the perceived approachability of ophthalmologists and level of prestige associated with a career in ophthalmology. Previously, ophthalmology has held a significant gender imbalance, as demonstrated by the 75% male predominance amongst all ophthalmologists. However, ophthalmologists under 35 are closer to an even gender distribution according to recent CMA data [15].

Our survey results demonstrated a more favourable opinion of ophthalmology as a profession among students with prior exposure to the field. The differences are quite substantial between the two groups, with 11 of 36 questions (31%) registering significantly different responses. Of these differences, most occur within the domains of culture, scope of practice, and whether they enjoyed the subject material/physiological concepts related to ophthalmology. There is more to elucidate in future studies around exposure and there is ambiguity as to what a respondent considered as exposure to ophthalmology, aside from having a family member or friend practicing ophthalmology. It may be worth determining what types of exposures lead to the most positive perceptions in future studies. Interestingly, this finding overlaps with the results reached by Kim et al. in their assessment of urology [13].

The most intuitive of these differences is related to the subject material domain. Those who have had prior exposure would be more likely to form a working relationship with the concepts and physiology related to the field, which would naturally result in a stronger grasp of the relevant educational material during lecture/self-study. Furthermore, it was more likely their interest in the material has or will lead them to pursue an elective in the specialty, and better understand the role of ocular health as part of an individual’s overall wellness. Additionally, first-hand experience could reduce the opinions of the field based on unreliable sources of information about the profession.

These positive opinions were mirrored by the improved responses seen by those with family members or friends that are or were previously practicing ophthalmologists. Again, these individuals would have had exposure to the field, albeit indirectly, and would likely have a better understanding of ophthalmology as a profession. Therefore, promoting and increasing early exposure to ophthalmology among medical students would potentially improve overall interest in the field, or at the very least allow a better understanding of whether ophthalmology might be right for them.

A student’s interest in ophthalmology was another important factor that influenced one’s opinion of the field. Those interested in ophthalmology displayed higher scores for opinions of both patient relationships and patient diversity. Although these topics are subjective, it appears as though those interested in ophthalmology appreciated the patient populations that are typical in an ophthalmology practice and the physician-patient relationships formed as an ophthalmologist. The balance between OR and clinic time was another enticing aspect to those interested in ophthalmology and is supported by previous studies [16]. However, one must be particularly vigilant when interpreting these results as a type of confirmation bias may influence the rankings given from this cohort, given their interest in the field [17].

Given the subjectivity entrenched within many of the survey’s questions, only a subset of questions was selected to attain a more objective rating of ophthalmology from students (denoted with an asterisk in Tables 1 and 3). Regression analysis revealed the significance of interest in ophthalmology in having a positive view of ophthalmology as a career. Interest in ophthalmology increased the adjusted survey scores by 7% (*p* < 0.02) of the maximum possible score. This result is somewhat expected, as it is obvious that an interest in the subject would be cultivated by positive views of the career. Of note, the view of ophthalmology from all students surveyed is quite high. Future studies could focus on personality factors that might add to the understanding of an individual’s interest in ophthalmology.

Exposure to ophthalmology in medical school was felt to be lacking according to the survey. This coincided with the belief that ophthalmology topics taught in medical school were not applicable to the general practitioner in maintaining the overall health of patients. Previous studies have shown that ophthalmology teaching has been reduced significantly among Canadian medical schools [3, 18, 19]. Our survey demonstrates that students now feel that the current exposure is inadequate. Furthermore, the survey emphasized that the current medical curriculum fails to demonstrate the importance of eye health, and its role in the overall health of patients [18, 19]. Increasing exposure to ophthalmology would likely negate the current deficiencies perceived by medical students and allow students to appreciate the importance of ocular health in the determination and maintenance of appropriate general health and quality of life [20, 21].

This survey provides an understanding of how medical students at the Schulich School of Medicine and Dentistry at Western University (London, Ontario) feel about Ophthalmology as a career. While we believe that our survey accurately demonstrates opinions of ophthalmology among medical students, there are limitations of this study that must be addressed. One potential limitation of this study is the single center population sample. This limits potential applications to schools across Canada, and medical education in general. However, similarly to the Schulich School of Medicine and Dentistry, many medical schools in Canada have limited their ophthalmology teaching in recent years and do not have compulsory ophthalmology electives, especially among medical schools with 3-year undergraduate medical programs. Therefore, many of the observations from the survey should apply to other medical schools in Canada. In the future, the distribution of similar surveys to multiple centers may address these concerns. Additionally, it should be noted we are operating on an assumption that the surveys, while subjective and based on opinion, were completed truthfully and accurate to the respondent’s true beliefs.

Furthermore, the 20% response rate was lower than expected and should be considered as a limitation of this study. This may be explained by the potential low level of interest in ophthalmology-related surveys among medical students, or due to the abundance of requests for research participants directed at medical students. It should also be noted that the subject matter of the survey may have attracted students who have an above-average interest in ophthalmology, while those without interest may have ignored it. If we could entice every single medical student to complete the survey, we may have found differing results. However, this is a limitation inherent to all surveys sent out to a particular population. As the data exist, there may have been some degree of selection bias in the results. Regardless, the 135 received responses are still well within the recommended 10 participants for each independent variable [22].

## Conclusions

In conclusion, our survey has demonstrated that medical students at Schulich have a positive view of ophthalmology as a specialty. Medical students had a less favourable view about the perceived limited range of practice, low inpatient to outpatient ratio, limited interaction with other specialties, and the lack of job opportunities available in the field. The highest scoring domains included perceptions of favourable lifestyle, high flexibility, and the positive impact ophthalmologists have on their patient’s quality of life. Furthermore, the amount of exposure to ophthalmology in medical school was rated unfavourably, as was the perceived applicability of learned material. These results suggest that some changes to the medical curriculum may be required.

The characteristics related to the most positive views of ophthalmology were the year of medical training, exposure to ophthalmology, and interest in ophthalmology. An individual’s interest in ophthalmology appears to be the strongest variable inherent to the positive views of the specialty. Future updates to the medical curriculum should focus on correcting potential misconceptions of ophthalmology early during medical school training to allow the best-suited candidates the opportunity to cultivate interest and appropriate perceptions of ophthalmology. If the best-suited candidates were directed to ophthalmology sooner, they would have greater opportunity to develop a well-rounded CV for their CaRMS application. With better matching between candidates and specialties, it is possible to envision more job satisfaction as practicing physicians in the long term. Looking beyond ophthalmology, while our findings cannot be generalized to other specialties, our methodology could be used to determine medical students’ perceptions of those domains in terms of education and practice outcomes.

## Data Availability

The datasets used and/or analysed during the current study are available from the corresponding author on reasonable request.

## References

[1] Yang Y, Li J, Wu X (2019). Factors influencing subspecialty choice among medical students: a systematic review and meta-analysis. BMJ Open.

[2] R-1 match interactive data - CaRMS. https://www.carms.ca/en/data-and-reports/r-1-match-interactive-data/. Published 2017. Accessed 21 Aug 2017.

[3] Shah M, Knoch D, Waxman E (2014). The state of ophthalmology medical student education in the United States and Canada, 2012 through 2013. Ophthalmology..

[4] Noble J, Somal K, Gill HS, Lam W-C (2009). An analysis of undergraduate ophthalmology training in Canada. Can J Ophthalmol.

[5] Wright B, Scott I, Woloschuk W, Brenneis F (2004). Career choice of new medical students at three Canadian universities: Family medicine versus specialty medicine. CMAJ..

[6] Erzurum VZ, Obermeyer RJ, Fecher A (2000). What influences medical students’ choice of surgical careers. Surgery..

[7] Scott IM, Matejcek AN, Gowans MC, Wright BJ, Brenneis FR (2008). Choosing a career in surgery: factors that influence Canadian medical students’ interest in pursuing a surgical career. Can J Surg.

[8] Succar T, Zebington G, Billson F (2013). The impact of the virtual ophthalmology clinic on medical students’ learning: a randomised controlled trial. Eye..

[9] Al-Najmi YA, Subki AH, Alzaidi NS (2021). Medical schools’ ophthalmology course: an appraisal by ophthalmology residents. Int J Gen Med.

[10] Nguyen AX, Pur DR, Lo C, Gottlieb C, Hardy I (2021). 2020–2021 Canadian Ophthalmology Student Interest Group. Experiences from a national webinar with recently matched Canadian ophthalmology residents for medical students [published online ahead of print, 2021 Nov 14]. Can J Ophthalmol.

[11] Linz MO, Jun AS, Clever SL, Lawson SM, Sanyal A, Scott AW (2018). Evaluation of medical students' perception of an ophthalmology career. Ophthalmology..

[12] Scott I, Gowans M, Wright B, Brenneis F, Banner S, Boone J. Determinants of choosing a career in family medicine. CMAJ. 2011;183(1). 10.1503/cmaj.091805. 10.1503/cmaj.091805 PMC3017271 20974721

[13] Kim S, Farrokhyar F, Braga LH (2016). Survey on the perception of urology as a specialty by medical students. J Can Urol Assoc.

[14] 2014 Results for Surgical Specialists by specialty. - National Physician Survey. http://nationalphysiciansurvey.ca/result/2014-results-surgical-specialists-specialty/. Published 2014. Accessed 21 Aug 2017.

[15] Canadian Specialty Profiles - Ophthalmology. https://www.cma.ca/En/Pages/specialty-profiles.aspx. Published 2016. Accessed 21 Aug 2017.

[16] Yousuf SJ, Kwagyan J, Jones LS (2013). Applicants’ choice of an ophthalmology residency program. Ophthalmology..

[17] Nickerson RS (1998). Confirmation bias: A ubiquitous phenomenon in many guises. Rev Gen Psychol.

[18] Clarkson JG (2003). Training in Ophthalmology Is Critical for All Physicians. Arch Ophthalmol.

[19] Stern GA. Teaching ophthalmology to primary care physicians. The Association of University Professors of Ophthalmology Education Committee. Arch Ophthalmol (Chicago, Ill 1960). 1995;113(6):722–724. http://www.ncbi.nlm.nih.gov/pubmed/7786211. 10.1001/archopht.1995.01100060048029 7786211

[20] Chia EM, Wang JJ, Rochtchina E, Smith W, Cumming RR, Mitchell P (2004). Impact of Bilateral Visual Impairment on Health-Related Quality of Life: The Blue Mountains Eye Study. Investig Ophthalmol Vis Sci.

[21] Ivers RQ, Cumming RG, Mitchell P, Attebo K (1998). Visual impairment and falls in older adults: the Blue Mountains Eye Study. J Am Geriatr Soc.

[22] Vittinghoff E, McCulloch CE. Relaxing the rule of ten events per variable in logistic and cox regression. Am J Epidemiol. 2007. 10.1093/aje/kwk052. 10.1093/aje/kwk052 17182981

